# The Globe Microscope

**Published:** 1872-07

**Authors:** 


Globe Microscope.—We omitted to state in our notice of the Globe Microscope in our last number that they could be procured in this city of Mr. Wm. Peabody for $2.50 each.

